# An inducible RIPK3-driven necroptotic system enhances cancer cell–based immunotherapy and ensures safety

**DOI:** 10.1172/JCI181143

**Published:** 2024-11-19

**Authors:** Kok-Siong Chen, Sarah Manoury-Battais, Nobuhiko Kanaya, Ioulia Vogiatzi, Paulo Borges, Sterre J. Kruize, Yi-Ching Chen, Laura Y. Lin, Filippo Rossignoli, Natalia Claire Mendonca, Khalid Shah

**Affiliations:** 1 Center for Stem Cell and Translational Immunotherapy and; 2 Department of Neurosurgery, Brigham and Women’s Hospital, Harvard Medical School, Boston, Massachusetts, USA.; 3 Department of Education and Research in Biology, ENS Paris-Saclay, Université Paris-Saclay, Gif-sur-Yvette, France.; 4 Harvard Stem Cell Institute, Harvard University, Cambridge, Massachusetts, USA.

**Keywords:** Immunology, Oncology, Brain cancer, Cancer immunotherapy, Translation

## Abstract

Recent progress in cancer cell–based therapies has led to effective targeting and robust immune responses against cancer. However, the inherent safety risks of using live cancer cells necessitate the creation of an optimized safety switch without hindering the efficacy of immunotherapy. The existing safety switches typically induce tolerogenic cell death, potentially leading to an immunosuppressive tumor immune microenvironment (TIME), which is counterproductive to the goals of immunotherapy. Here, we developed and characterized an inducible receptor-interacting protein kinase 3–driven (RIPK3-driven) necroptotic system that serves a dual function of safety switch as well as inducer of immunogenic cell death, which in turn stimulates antitumor immune responses. We show that activation of the RIPK3 safety switch triggered immunogenic responses marked by an increased release of ATP and damage-associated molecular patterns (DAMPs). Compared with other existing safety switches, incorporating the RIPK3 system inhibited tumor growth, improved survival outcomes in tumor-bearing mice, and fostered long-term antitumor immunity. Moreover, the RIPK3 system reinvigorated the TIME by promoting DC maturation, polarizing the macrophages toward a M1 phenotype, and reducing the exhaustion of CD4^+^ and CD8^+^ T lymphocytes. Our study highlights the dual role of the RIPK3-driven necroptotic system in improving the safety and efficacy of cancer cell–based therapy, with broader implications for cellular therapies.

## Abstract 2

We report the creation and characterization of an optimized inducible RIPK3-driven necroptotic system that has a dual function of providing safety and inducing tumor eradication and long-term immunity in highly immunosuppressive tumors.

## Introduction

Therapeutic tumor cells hold promise as anticancer agents because of their ability to serve as the natural source of neoantigens and the ease of being genetically engineered. We recently developed cancer cell–based therapies using live tumor cells that not only target tumor cells directly but also potentiate the immune system’s response, leading to immune-mediated cell death (1). To heighten the safety of this therapeutic approach, it is necessary to incorporate suicide systems or kill switches. The commonly used safety switches — herpes simplex virus thymidine kinase (HSV-TK) (2), inducible caspase 9 (iCasp) (3), and the CD20/rituximab system (4) — typically induce a tolerogenic form of cell death that may lead to an immunosuppressive tumor microenvironment (TME) (5). These attributes of existing safety switches inadvertently oppose the goals of immunotherapy by dampening the desired immune response against cancer cells. Given these limitations, there is an urgent need to explore better suicide systems.

One such approach involves the use of the inducible immunogenic cell death (ICD) suicide system. Characterized by the exposure of damage-associated molecular patterns (DAMPs) and a cascade of intracellular events leading to robust antitumor immunity (6–8), ICD distinguishes itself from other types of cell death by its capacity to stimulate a potent immune response against tumor cells. Unlike apoptosis, a noninflammatory form of cell death, necroptosis is a programmed cell death that leads to the release of DAMPs, which can stimulate an immune response (9–13). Specifically, necroptosis occurs downstream of the receptor-interacting protein kinases 1 and 3 (RIPK1 and RIPK3) (14). These proteins oligomerize through their RIP homotypic interaction motif (RHIM) to form the necrosome together with mixed lineage kinase domain–like protein (MLKL), thereby inducing necroptosis (14). Throughout the whole process, RIPK3 homodimerization and phosphorylation are the key events triggering the immunogenic cell death of necroptosis (15). Leveraging this phenomenon in the context of a suicide system could therefore provide dual benefits: safety control and potential therapeutic augmentation.

Recognizing the specific need for a dual system that allows immunogenic cell killing and a reliable kill switch in cancer cell–based immunotherapies, we developed and evaluated an inducible RIPK3-driven necroptotic suicide system using a whole tumor cancer cell vaccine as a therapeutic to treat glioblastoma (GBM). We first devised the switch by optimizing previously published designs of RIPK3 dimerization constructs (16, 17), which were utilized to study the necroptotic pathway. Our aim was to ensure that the necroptotic suicide mechanism is independent of RIPK1 dimerization, thereby avoiding caspase 8 negative regulation. This design allows for the conditional dimerization and activation of the RIPK3 protein upon exposure to a synthetic dimerizing drug. Next, we tested the activity of the safety switch by introducing the gene into GBM tumor cells and further evaluated the immunogenicity induced by the cell death, both in vitro and in vivo, in comparison with existing safety switches. Our goal was to evaluate the unique in vivo immune responses elicited by this approach in combination with cancer cell–based therapy. Our findings reveal that the RIPK3-driven necroptotic suicide system has an immense potential to combine safety with an enhanced therapeutic effect by improving antitumor immune responses.

## Results

### Robust necroptotic signaling correlates with improved therapeutic outcomes for patients with cancer.

**Figure 1:**
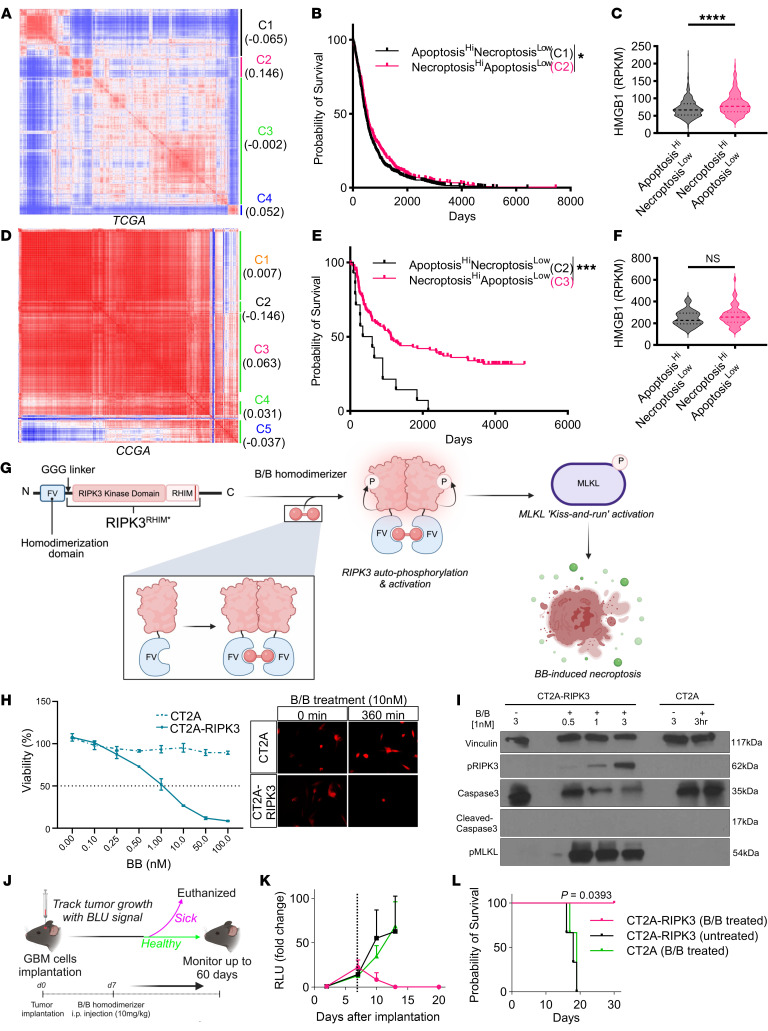
Establishing an inducible RIPK3-driven necroptotic safety switch. (**A**) Coclustering of primary tumor samples (*n* = 2,785) in TCGA based on apoptotic (GO: 0097190) and necroptotic (GO: 0097527) signaling pathway gene signatures. (**B**) Survival curves showing survival probability for C1 and C2 clusters from **A**. The survival curves between 2 groups were compared using a log-rank (Mantel-Cox) test with Bonferroni correction. (**C**) Expression of HMGB1 in C1 and C2 clusters from **A**. (**D**) Coclustering of primary tumor samples (*n* = 222) in the CCGA based on apoptotic (GO: 0097190) and necroptotic (GO: 0097527) signaling pathway gene signatures. (**E**) Survival curves showing overall survival probability for C2 and C3 clusters from **D**. The survival curves between 2 groups were compared using a log-rank (Mantel-Cox) test with Bonferroni correction. (**F**) Expression of HMGB1 in C2 and C3 clusters from **D**. (**G**) Schematic representation of the RIPK3 B/B-inducible safety switch system. (**H**) CT2A and CT2A-RIPK3 cell viability assay following treatment with B/B in a dose-dependent manner for 6 hours and time-lapse imaging of the cells before and after treatment with 10 nM B/B for 6 hours. (**I**) Western blot analysis of p-MLKL, p-RIPK3, and cleaved caspase 3 levels upon B/B treatment in CT2A and CT2A-RIPK3 cells with different treatment durations. (**J**) Schematic of the experimental timeline for intracranial GBM cell implantation and the treatment schedule for B/B. (**K**) Graph of Fluc signal in C57BL6 mice after intracranial implantation of CT2A cells with B/B treatment (*n* = 3) and CT2A-RIPK3 cells with (*n* = 3) and without (*n* = 3) B/B treatment. Dotted line represents the B/B treatment time point. Data represent the mean ± SEM. (**L**) Kaplan-Meier curves showing the survival probability for C57BL6 mice after intracranial implantation with CT2A with B/B treatment (*n* = 3) and CT2A-RIPK3 with (*n* = 3) and without (*n* = 3) B/B treatment. *P < 0.05, ***P < 0.001, and ****P < 0.0001, by log-rank (Mantel-Cox) test with Bonferroni correction (**B**, **E**, and **L**) and unpaired, 2-tailed Student’s *t* test (**C** and **F**). RPKM, reads per kilobase per million mapped reads.

To understand how necroptotic signaling within the TME affects the patients’ clinical outcome, primary tumor samples in The Cancer Genome Atlas (TCGA) dataset were coclustered on the basis of both Gene Ontology (GO) apoptotic (GO: 0097190) and necroptotic (GO: 0097527) signaling pathway gene signatures, and 4 clusters were generated (Figure 1A and Supplemental Table 1; supplemental material available online with this article; https://doi.org/10.1172/JCI181143DS1). Based on the combined gene score (upregulation of necroptotic signaling and downregulation of apoptotic signaling) for each cluster, cluster 2 (C2) was associated with the most robust necroptotic signaling (necroptosis^hi^) and low apoptotic signaling (apoptosis^lo^), whereas C1 had the lowest score for necroptotic signaling (necroptosis^lo^) but the highest apoptotic signaling (apoptosis^hi^) (Figure 1A and Supplemental Table 1). Importantly, necroptosis^hi^apoptosis^lo^, as reflected in C2, was associated with an improved clinical outcome and a significantly better prognosis compared with C1, which was apoptosis^hi^necroptosis^lo^ (Figure 1B and Supplemental Table 1). We also confirmed that C2 samples had significantly higher expression of high-mobility group box 1 (HMGB1), a typical DAMP that is released during necroptosis (18) (Figure 1C). A similar analysis (Figure 1D and Supplemental Table 2) using a public mRNA-Seq dataset of CCGA glioma patients (19) demonstrated that C3 (necroptosis^hi^apoptosis^lo^) is associated with a better survival probability compared with C2 (apoptosis^hi^necroptosis^lo^) (Figure 1E and Supplemental Table 2), but we observed no significant difference in HMGB1 expression (Figure 1F). These findings suggest that upregulation of necroptotic signaling and downregulation of apoptotic signaling within the TME is likely to improve clinical outcomes for patients, highlighting the association of necroptotic signaling in shaping a more immune-active TME.

### Generation of engineered tumor cells with an inducible RIPK3-driven necroptotic safety switch.

To establish an inducible RIPK3-driven necroptotic suicide system, a murine RIPK3 cDNA fragment encoding FKBP^F36V^ and mutated RHIM domains were subcloned into a third-generation lentiviral transfer vector based on previously published RIPK3 dimerization constructs (16, 17) (Figure 1G). For enhanced specificity, we opted to use a mutated form of FKBP rather than the WT protein (20). The cDNA fragment for the FKBP^F36V^ domain was fused to the N-terminal of RIPK3 to allow autophosphorylation and activation of RIPK3 protein upon homodimerization (16, 17), thus activating necroptosis. The RHIM domain was mutated to prevent the interaction of RIPK3 with endogenous RIPK1, thereby avoiding RHIM-dependent necrosome formation and subsequent regulation by caspase 8 (16, 17). This system allows the inducible intracellular homodimerization of RIPK3 proteins upon administration of AP20187 (B/B homodimerizer, hereafter referred to as B/B), a rapamycin analog.

To test the safety switch in vitro, we transduced CT2A, an immune-suppressive syngeneic GBM cell line (21), with the RIPK3 lentiviral construct, resulting in the engineered CT2A cell line (hereafter referred to as CT2A-RIPK3). Compared with WT CT2A, the viability of CT2A-RIPK3 cells was reduced upon B/B treatment in a dose-dependent and time-dependent manner (Figure 1H). Moreover, Western blot analysis of cell lysates of B/B-treated CT2A-RIPK3 cells showed phosphorylation of MLKL and RIPK3 (p-MLKL and p-RIPK3) but no cleaved caspase 3 expression (Figure 1I and Supplemental Figure 4), confirming the activation of necroptotic signaling, and the cell death observed was not apoptosis driven. To validate the inducible suicide system of the engineered tumor cells in vivo, CT2A and CT2A-RIPK3 tumor cells were intracranially implanted into immunocompetent C57BL6 mice (Figure 1J). Bioluminescence Fluc imaging (BLI) revealed aggressive tumor growth for all mice implanted with CT2A and CT2A-RIPK3 cells (Figure 1K). Upon intraperitoneal administration of B/B, we observed complete tumor eradication in CT2A-RIPK3 tumor–bearing mice (Figure 1K). Furthermore, the tumor-free mice survived until the termination of the experiment (Figure 1L), confirming the effectiveness of the inducible RIPK3 safety switch in tumor cells in vivo.

### Cell death induced by the RIPK3 safety switch is more immunogenic than RapaCas9 or HSV-TK systems in vitro.

**Figure 2:**
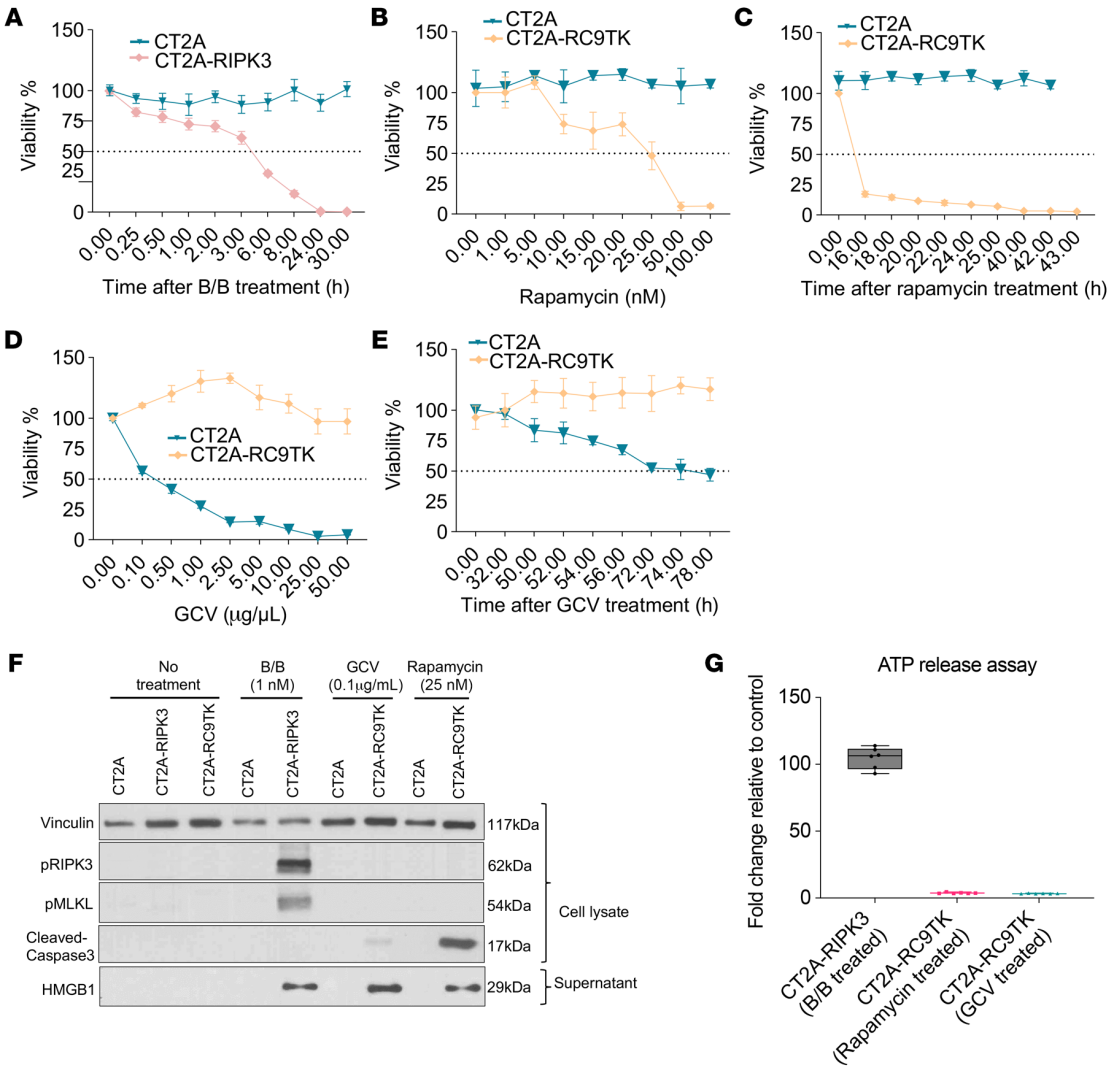
Cell death induced by the RIPK3 safety switch is more immunogenic than the RapaCas9 or HSV-TK system in vitro. (**A**) CT2A and CT2A-RIPK3 cell viability assay following treatment with 1 nM B/B for different durations. (**B**) CT2A and CT2A-RC9TK cell viability assay upon treatment with rapamycin in a dose-dependent manner for 24 hours. (**C**) CT2A and CT2A-RC9TK cell viability assay following treatment with 25 nM rapamycin for different durations. (**D**) CT2A and CT2A-RC9TK cell viability assay following treatment with GCV in a dose-dependent manner for 72 hours. (**E**) CT2A and CT2A-RC9TK cell viability assay upon treatment with 0.1 μg/mL GCV for different durations. (**F**) Western blot analysis of p-MLKL, p-RIPK3, cleaved caspase 3, and HMGB1 levels following treatment or without B/B in CT2A and CT2A-RIPK3 cells and with or without treatment with rapamycin or GCV in CT2A and CT2A-RC9TK cells, based on the respective EC_50_ dose and duration. (**G**) Box plot of ATP release assay following treatment with or without B/B in CT2A and CT2A-RIPK3 cells or treatment with or without rapamycin or GCV in CT2A and CT2A-RC9TK cells, based on the respective EC_50_ dose and duration.

Recently, we developed a 2-layered safety switch that comprises RapaCas9 and HSV-TK suicide systems for safer cell therapies (22). Although these systems have been tested extensively in vitro and in vivo (1, 22), the immunogenicity induced by cell death has not to our knowledge been investigated. Given that the release of ATP from dying cells and the postmortem release of HMGB1 into the extracellular space are 2 of the 3 major hallmarks of ICD (23), we sought to compare the cell death immunogenicity of these 3 safety switches on the basis of these 2 measures. As such, we engineered CT2A to express RapaCas9TK using the LV-RapaCas9TK construct (22) (hereafter referred to as CT2A-RC9TK). For a fair comparison between these 3 safety switches, we first determined their EC_50_ with different doses of the drugs and the durations of the treatment. The EC_50_ for B/B to induce cell death in CT2A-RIPK3 cells was 1 nM (Figure 1H) after 5 hours of treatment (Figure 2A). To induce CT2A-RC9TK cell death, the EC_50_ for rapamycin (RapaCas9 system) was 25 nM (Figure 2B) after 9 hours of treatment (Figure 2C) and 0.1 μg/mL for ganciclovir (GCV) (HSV-TK system) (Figure 2D) after 72 hours of treatment (Figure 2E). Western blot analysis for the lysates of CT2A-RIPK3 cells treated with B/B showed phosphorylation of MLKL and RIPK3 but not in CT2A-RC9TK cells treated with rapamycin or GCV (Figure 2F). On the other hand, we detected cleaved caspase 3 expression in the lysates of CT2A-RC9TK cells treated with GCV or rapamycin but not in other groups (Figure 2F and Supplemental Figure 5), confirming the induction of caspase-dependent apoptosis. Next, we confirmed HMGB1 expression in the supernatant of B/B-treated CT2A-RIPK3 cells and GCV- and rapamycin-treated CT2A-RC9TK cells (Figure 2F). Furthermore, we observed the highest fold change of the ATP release relative to the control in supernatant collected at EC_50_ for B/B-treated CT2A-RIPK3 cells compared with the other 2 suicide systems (Figure 2G). These findings reveal that the cell death induced by the RIPK3 safety switch was necroptotic and more immunogenic.

### Tumor cell death induced by the RIPK3 safety switch elicits long-term immunity in vivo.

**Figure 3:**
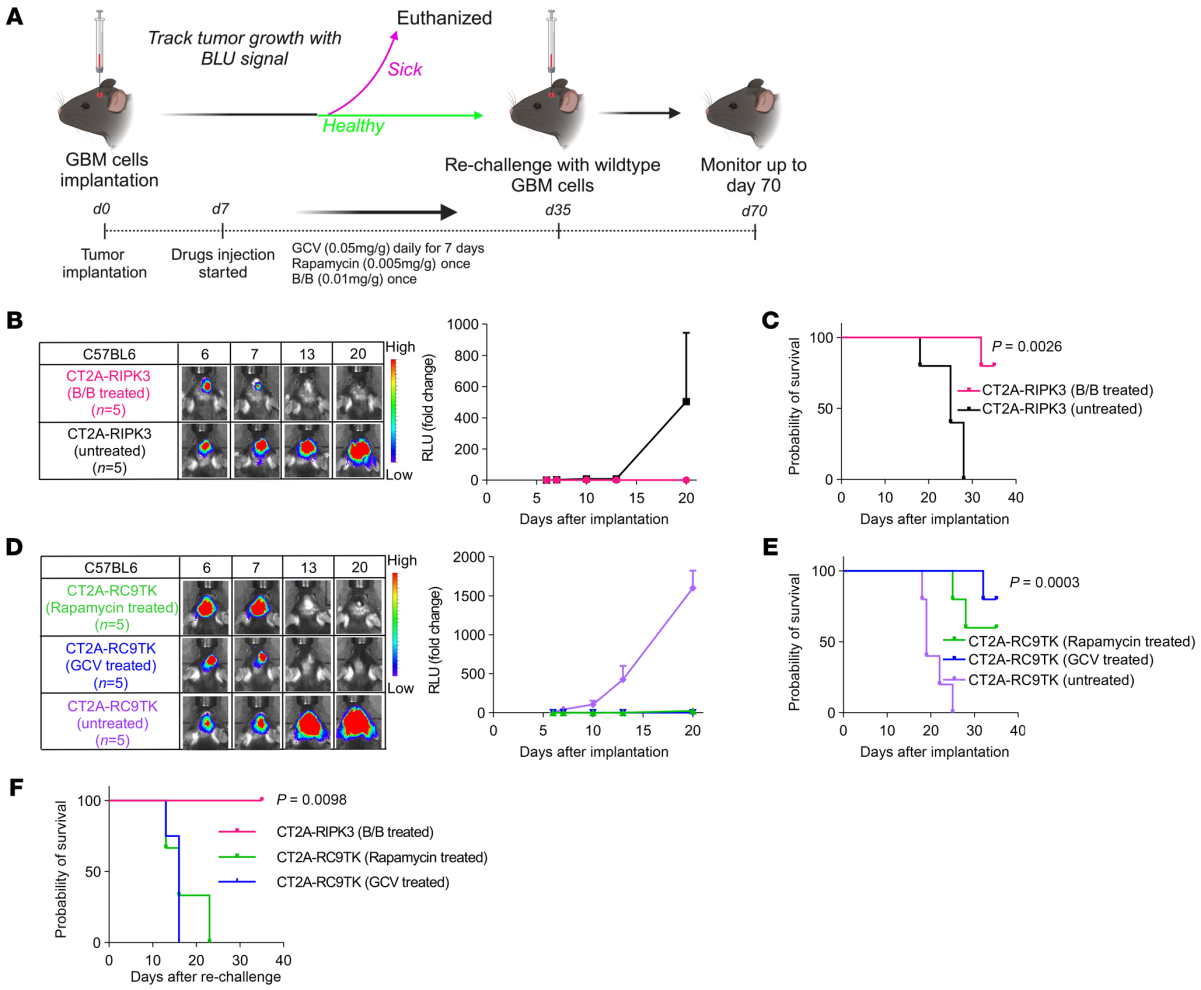
Tumor cell death induced by the RIPK3 safety switch elicits long-term immunity and antitumor therapeutic benefits in vivo. (**A**) Schematic of the experimental timeline for intracranial GBM coimplantation and the schedule for B/B, rapamycin, or GCV treatment. (**B**) Representative images and graph of Fluc signal in C57BL6 mice after intracranial implantation with CT2A-RIPK3 cells, with (*n* = 5) and without (*n* = 5) B/B treatment. Data represent the mean ± SEM. (**C**) Kaplan-Meier curves demonstrating the survival probability of C57BL6 mice after intracranial implantation with CT2A-RIPK3 cells with (*n* = 5) and without (*n* = 5) B/B treatment. Data were assessed by log-rank (Mantel-Cox) test with Bonferroni correction. (**D**) Representative images and graph of Fluc signal in C57BL6 mice after intracranial implantation with CT2A-RC9TK cells with rapamycin treatment (*n* = 5) or GCV treatment (*n* = 5) and without any treatment (*n* = 5). Data represent the mean ± SEM. (**E**) Kaplan-Meier curves demonstrating the survival probability of C57BL6 mice after intracranial implantation of CT2A-RC9TK cells with rapamycin treatment (*n* = 5) or GCV treatment (*n* = 5) and without any treatment (*n* = 5). Data were assessed by log-rank (Mantel-Cox) test with Bonferroni correction. (**F**) Kaplan-Meier curves demonstrating the survival probability of the surviving C57BL6 mice from **C** and **E** after rechallenge with parental CT2A cells. Data were assessed by log-rank (Mantel-Cox) test with Bonferroni correction (**C**, **E**, and **F**).

To investigate the cell death immunogenicity for each safety switch in vivo, CT2A-RIPK3 and CT2A-RC9TK cells were intracranially implanted into immunocompetent C57BL6 mice (Figure 3A). The intracranial tumors of 4 of 5 CT2A-RIPK3 tumor–bearing mice were completely eradicated upon treatment with B/B on day 7 after tumor implantation (Figure 3B). These mice remained tumor free and survived beyond 30 days (Figure 3C). For CT2A-RC9TK cells, 4 of 5 tumor-bearing mice treated with GCV on day 7 after tumor implantation had complete tumor eradication (Figure 3D), and these mice remained tumor free and survived beyond 30 days (Figure 3E). For CT2A-RC9TK tumor–bearing mice treated with rapamycin, 3 of 5 of them had complete tumor eradication (Figure 3D), and these mice also remained tumor free and survived beyond 30 days (Figure 3E). To test the immunization effects of these suicide systems, we next rechallenged the mice surviving from the first implantation on day 35 after tumor implantation with the parental CT2A cells in the contralateral brain hemisphere (Figure 3A). The median survival for the mice from both the RapaCas9 and HSV-TK groups was 15 days after rechallenge, whereas all mice from the group subjected to the RIPK3 system survived until the termination of the experiment, and no tumor growth was observed (Figure 3F). These findings indicate that tumor cell death induced by RIPK3 safety switch can elicit long-term antitumor immunity in mice.

### Cell death induced by the RIPK3 safety switch inhibits tumor growth of parental CT2A cells and improves survival outcomes by reinvigorating the tumor immune microenvironment in vivo.

**Figure 4:**
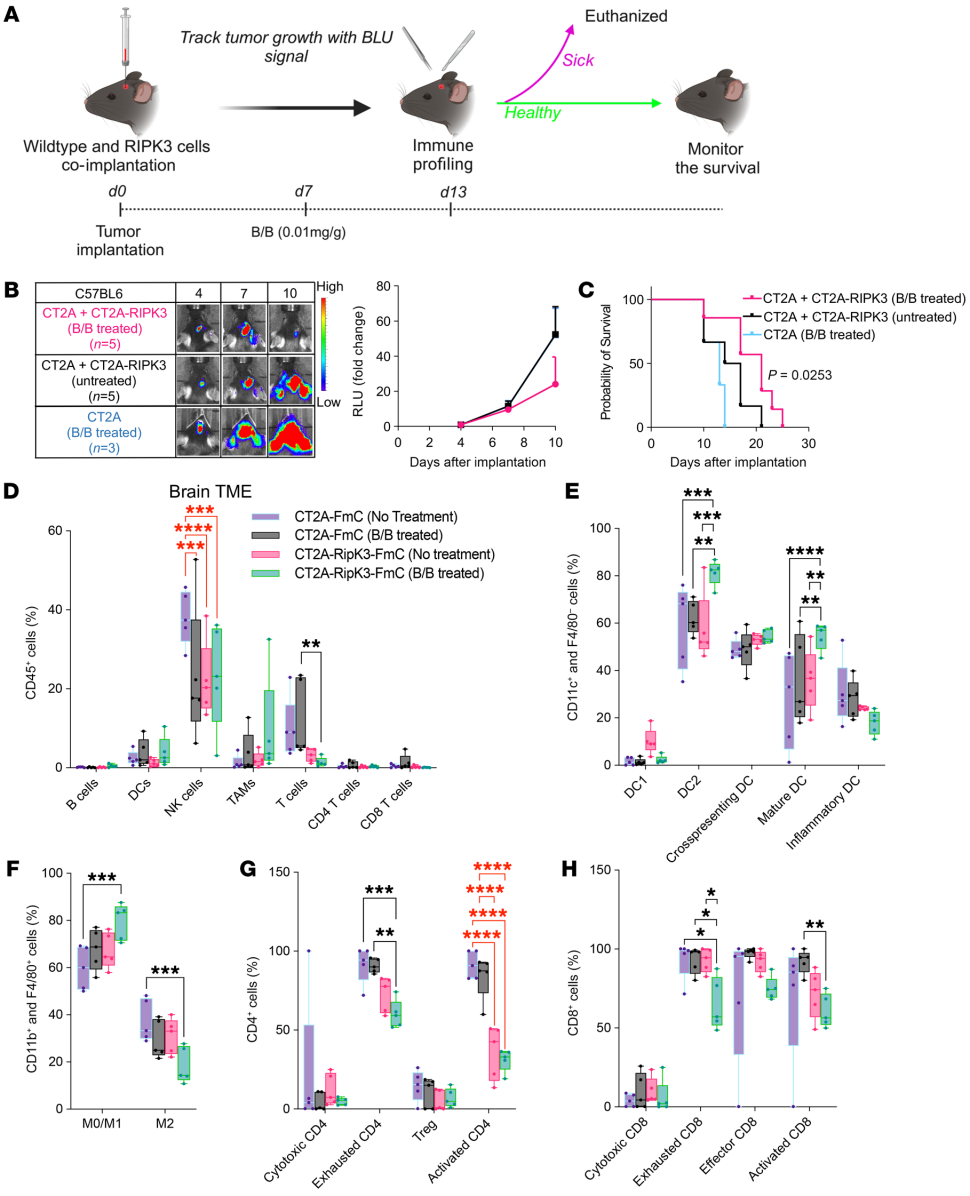
Cell death induced by the RIPK3 safety switch inhibits tumor growth and reinvigorates the TIME in vivo. (**A**) Schematic of the experimental timeline for intracranial mixed GBM cell implantation and B/B treatment schedule and the tissue collection time points for immune profiling. (**B**) Representative images and graph of Fluc signal in C57BL6 mice after intracranial implantation of 150,000 CT2A cells with B/B treatment (*n* = 3) and a mixture of 75,000 CT2A cells and 75,000 CT2A-RIPK3 cells with (*n* = 5) or without (*n* = 5) B/B treatment. Data represent the mean ± SEM. (**C**) Kaplan-Meier curves demonstrating the survival probability of C57BL6 mice after intracranial implantation of CT2A cells with B/B treatment (*n* = 3) and a mixture of CT2A and CT2A-RIPK3 cells with (*n* = 5) or without (*n* = 5) B/B treatment. Data were assessed by log-rank (Mantel-Cox) test with Bonferroni correction. (**D**) Box plot showing immune profiling of tumor tissues harvested from the mouse brain 5 days after prodrug treatment for the groups of mice implanted with CT2A cells with (*n* = 5) or without (*n* = 5) B/B treatment and the groups mice implanted with a mixture of CT2A and CT2A-RIPK3 cells with (*n* = 5) or without (*n* = 5) B/B treatment. (**E**) Box plot showing the profile of DC subpopulations from the experiment in **D**. (**F**) Box plot showing the profile of the tumor-associated macrophage (TAM) subpopulations from the experiment in **D**. (**G**) Box plot showing the profile of CD4^+^ T cell subpopulations from the experiment in **D**. (**H**) Box plot showing the profile of CD8^+^ T cell subpopulations from the experiment in **D**. The whiskers represent the minimum to the maximum. Data were analyzed by 2-Way ANOVA with the 2-stage step-up method of Benjamini, Krieger, and Yekutieli for multiple-comparison correction (**D**–**H**). *P < 0.05, **P < 0.01, ***P < 0.001, and ****P < 0.0001. The black significance line represents changes due to activation of the RIPK3 suicide system, and the red significance line represents changes due to differences in cell type or drug.

To determine whether the cell death induced by the RIPK3 suicide system has potential therapeutic augmentation, we coimplanted WT CT2A cells and CT2A-RIPK3 cells intracranially into C57BL6 mice (Figure 4A). The tumor growth of the parental CT2A cells was inhibited upon activation of the RIPK3 suicide system compared with the controls (Figure 4B). Moreover, these mice also had a significantly better survival outcome compared with the controls (Figure 4C). These findings imply that the application of the RIPK3 safety switch has antitumor therapeutic benefits.

To assess the effect of the RIPK3 system on the tumor immune microenvironment (TIME), we analyzed the immune profile (Supplemental Figure 1) after activation of the safety switch in vivo (Figure 4A). Control groups were used to ensure that the observed changes were due to induced cell death and not just the presence of different cell types or the drug. Overall, we observed reduced T cell numbers in the TME (Figure 4D). Although the relative number of DCs remained unchanged (Figure 4D), the number of DC2 and mature DCs significantly increased (Figure 4E), suggesting that the cell death induced by the RIPK3 safety switch promoted DC maturation and DC2-mediated immune responses. In addition, we observed an increased polarization of macrophages toward the M1 phenotype (Figure 4F), implying the presence of an increased inflammatory factor within the TIME. For T lymphocytes, a significant reduction of exhausted CD4 (Figure 4G) and CD8 (Figure 4H) lymphocytes, as well as activated CD8 lymphocytes (Figure 4H), was observed. These findings indicate a more immune-reactive environment after RIPK3 safety switch activation.To provide insights into potential differences in immune responses between safety switches, we also analyzed the immune profile of the TIME after activation of RapaCas9 or HSV-TK. Unlike RIPK3, activation of RapaCas9 and HSV-TK did not change the overall immune cell population (Supplemental Figure 2A) or the subpopulations of DCs, macrophages, and CD4 lymphocytes (Supplemental Figure 2, B–D), but we observed a significant reduction of exhausted, effector, or activated CD8 lymphocytes for RapaCas9 activation (Supplemental Figure 2E).

Considering the importance of tumor-draining lymph nodes (TDLNs) for antitumor immune responses (24), we assessed the effect of RIPK3 safety switch activation on the TDLN. We observed an increase in DCs and NK cells (Supplemental Figure 3A) and a significant reduction in DC2 cells (Supplemental Figure 3B). Yet, we observed no significant changes in CD4 (Supplemental Figure 3C) or CD8 (Supplemental Figure 3D) lymphocytes. These findings corroborate the observation that cell death induced by the RIPK3 safety switch reinvigorated the TIME and induced a more robust immune response than the other 2 systems.

### The RIPK3 safety switch synergistically enhances the therapeutic efficiency of cancer cell–based immunotherapy and promotes long-term immunity.

**Figure 5:**
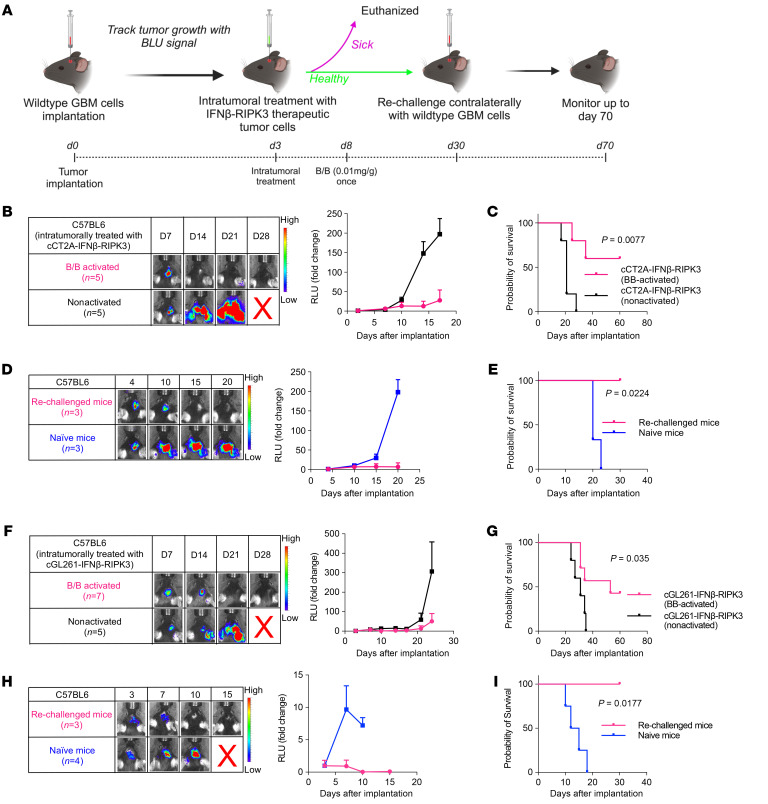
Tumor cell death induced by the RIPK3 safety switch synergistically improves the antitumor therapeutic benefits of cancer cell–based therapy and elicits long-term immunity in vivo. (**A**) Schematic of the experimental timeline for intracranial GBM implantation, the cCT2A–IFN-β–RIPK3 treatment schedule, and activation of the RIPK3 suicide system via B/B administration. (**B**) Representative images and graph of Fluc signal in C57BL6 mice following intracranial implantation of WT CT2A cells and intratumoral treatment with cCT2A–IFN-β–RIPK3 with (*n* = 5) or without (*n* = 5) B/B administration. (**C**) Kaplan-Meier curves demonstrating the survival probability of C57BL6 mice after intracranial implantation of WT CT2A cells and intratumoral treatment with cCT2A–IFN-β–RIPK3 with (*n* = 5) or without (*n* = 5) B/B administration. (**D**) Representative images and graph of Fluc signal after intracranial implantation with WT CT2A cells into the contralateral brain hemisphere of the surviving C57BL6 mice from **C** (*n* = 3) and naive mice (*n* = 3). (**E**) Kaplan-Meier curves demonstrating the survival probability of the surviving C57BL6 mice from **C** and naive mice after rechallenge with WT CT2A cells. (**F**) Representative images and graph of Fluc signal in C57BL6 mice after intracranial implantation of WT Gl261 cells and intratumoral treatment with cGL261–IFN-β–RIPK3 with (*n* = 7) or without (*n* = 5) B/B administration. (**G**) Kaplan-Meier curves demonstrating the survival probability of the C57BL6 mice after intracranial implantation of WT Gl261 cells and intratumoral treatment with cGL261–IFN-β–RIPK3 with (*n* = 7) or without (*n* = 5) B/B administration. (**H**) Representative images and graph of Fluc signal after intracranial implantation with WT GL261 cells into the contralateral brain hemisphere of the surviving C57BL6 mice from **G** (*n* = 3) and naive mice (*n* = 5). (**I**) Kaplan-Meier curves demonstrating the survival probability of the surviving C57BL6 mice from **G** and naive mice after rechallenge with WT GL261 cells. All data were assessed by log-rank (Mantel-Cox) test with Bonferroni correction and represent the mean ± SEM (**C**, **E**, **G**, and **I**).

To assess the synergistic therapeutic effect of the RIPK3 system and cancer cell–based therapy using live tumor cells, we sought to integrate this kill switch into the therapeutic tumor cells to treat established GBM tumors. The therapeutic tumor cells were derived from CT2A cells and genetically edited using CRISPR/Cas9 to knock out IFNAR1 and were engineered to express IFN-β (1). These modified cells, referred to as cCT2A–IFN-β cells, were shown not to grow in mice, as evidenced in our previous research (1). Furthermore, we engineered these cCT2A–IFN-β cells to include the RIPK3 safety switch, creating a new cell line named cCT2A–IFN-β–RIPK3. We treated established intracranial GBM tumors in mice intratumorally with cCT2A–IFN-β–RIPK3, with or without B/B activation of the RIPK3 safety switch (Figure 5A). Activation of the kill switch significantly improved survival, completely eradicating WT tumors in 3 of 5 mice, in contrast to the nonactivated group, in which all mice died (Figure 5, B and C). To study long-term immunity, surviving mice were rechallenged with WT CT2A cells implanted into the contralateral hemisphere of their brains, with naive mice serving as controls. Remarkably, we observed no tumor growth in the rechallenged mice (Figure 5D), and they survived this second challenge (Figure 5E). These results suggest that the RIPK3 system, when combined with cancer cell–based therapy, not only functions as a safety switch but also has a synergistic therapeutic effect and contributes to the development of adaptive immunity in mice.

To further validate the robustness and broad-spectrum applicability of the RIPK3 system and cancer cell–based therapy, we investigated another GBM tumor model, GL261. Similar to the CT2A model, Gl261 cells were genetically edited using CRISPR/Cas9 to knock out IFNAR1 and engineered to express IFN-β and the RIPK3 safety switch, creating the cGL261–IFN-β–RIPK3 cell line. We used these modified cells to treat established GL261 tumors in mice. Consistent with the results from the CT2A model, activation of the RIPK3 safety switch in the cGL261–IFN-β–RIPK3–treated mice significantly improved survival (Figure 5, F and G), and the fully treated mice survived the second challenge (Figure 5, H and I). The results from both tumor models support the conclusion that the RIPK3-driven necroptotic safety switch enhances the therapeutic efficacy of cancer cell–based therapies and promotes long-term immunity across different tumor models, thereby reinforcing the potential clinical applicability of this approach.

## Discussion

In this study, we repurposed and characterized a dual-function, inducible RIPK3-driven necroptotic suicide system that functions as a safety switch and can synergistically boost the efficiency of cancer cell–based immunotherapy, which in turn induces long-term immunity that prevents recurrence. Our findings revealed the potential of necroptotic signaling in shaping a more immune-active TIME, with the RIPK3 system showing a marked increase in ATP and HMGB1 release, indicating its heightened immunogenicity due to the induced cell death. Furthermore, the RIPK3 system demonstrated the ability to stimulate prolonged antitumor immunity in mice, inhibit tumor growth, and enhance survival outcomes.

Previous studies have shown that the introduction of ectopic necroptotic cells into the tumor milieu induces an antitumor response (13), but the design of the RIPK3 construct used may permit negative regulation by RIPK1 and caspase 8, which is undesirable in a safety switch context due to the risk of attenuating the necroptotic signal. Our studies extend these findings by addressing the safety concerns inherent in using live cancer cells for therapy. Our study not only reaffirms the beneficial role of the RIPK3 axis in initiating tumor immunity but also innovates it by presenting a dual-function necroptotic switch that mitigates the safety risks and elevates the immunotherapeutic potential of cancer cell–based therapies, providing a compelling case for clinical exploration.

In recent years, efforts have been made to repurpose tumor cells as carriers for delivering anticancer agents directly to primary tumor sites. Prominent strategies include using tumor cells as vehicles for oncolytic viruses (25, 26) and engineering them to secrete therapeutic agents targeting the tumor’s neovascular endothelium (27, 28). Typically, these cells are inactivated through lysis or irradiation before reintroduction into the body. This approach has been shown to trigger robust immune cell trafficking to the tumor site (29–32), resulting in the induction of an antitumor immune response in different cancer types in preclinical studies (33–37). Yet, clinical benefits in phase I–III clinical trials have been limited (38–40), likely because of a lack of active tumor-targeting ability of the lysed cells. Unlike inactivated tumor cells, living tumor cells inherently possess the ability to home in on and target tumor sites, making the use of engineered living tumor cells as therapeutics a logical step forward (1, 41). However, the safety concerns associated with this approach remain a major barrier to clinical translation. Therefore, the integration of an effective kill switch that does not compromise therapeutic efficacy is crucial. Our data suggest that the dual-function, RIPK3-driven necroptotic safety switch offers a promising avenue to address the safety concerns associated with these therapies as well as to synergistically enhance the therapeutic efficacy. The rationale behind introducing the tumor cells expressing IFN-β in our study (Figure 5) was to specifically assess the synergistic therapeutic effect when combined with the RIPK3 safety switch. Our earlier experiments (Figure 4) demonstrated therapeutic effects using CT2A cells without IFN-β modification, confirming that the RIPK3 safety switch functions independently of IFN-β signaling. The CT2A–IFN-β system allowed us to explore potential synergy without implying a dependence on IFN-β for the RIPK3 switch’s function, thereby reinforcing the versatility and robustness of our approach.

The necroptosis-based safety switch offers several advantages. The type of cell death induced by the safety switch plays a pivotal role in shaping the TIME (5). Unlike apoptosis, which is relatively less inflammatory (42), necroptosis resulted in the release of DAMPs and ATP (Figure 2, G and H), signifying the immunogenic nature of this type of cell death. The results from our TCGA dataset analysis further validated the importance of necroptotic signaling in improving clinical outcomes for patients with cancer. This underscores the potential of necroptosis in shaping a more immune-active TIME, which is critical for the success of immunotherapies (43).

In our in vitro and in vivo experiments, we observed that the engineered CT2A-RIPK3 cells, upon treatment with the synthetic dimerizing drug B/B, underwent necroptosis, thereby validating the functionality of this safety switch. The effectiveness of this system was further confirmed in vivo, where we observed complete tumor eradication in mice. Moreover, our study highlights the immunogenic nature of cell death induced by the RIPK3 system compared with other safety switches like RapaCas9 and HSV-TK. We believe this is an important finding, as it demonstrates the potential of the RIPK3 system to not only act as a safety switch but also to contribute to the induction of stronger immune responses for immunotherapy.

The ability of the RIPK3-driven safety switch to induce long-term immunity, as evidenced by our findings, marks a meaningful stride in cancer immunotherapy, especially in the context of tumor recurrence prevention. The observed changes in DC populations and T lymphocyte behavior after RIPK3 activation are particularly telling. The significant increase in mature DCs (Figure 4E) underscores a shift toward an environment conducive to enhanced antigen presentation, as mature DCs are more effective in presenting tumor antigens (44, 45), leading to a more potent and targeted immune response. We observed a diminished infiltration of total T cells (Figure 4D) following activation of the RIPK3 safety switch, which can be explained by the significant reduction in the exhausted CD4^+^ and CD8^+^ T cell population (Figure 4, G and H), a subset characterized by impaired function and reduced antitumor activity. Despite the limitations of our flow cytometry panel that included only programmed cell death 1 (PD-1) as a marker of exhaustion, the substantial reduction in PD-1^+^ cells suggests an overall shift toward a less exhausted, more functionally competent T cell phenotype. Additionally, the remaining CD8^+^ T cells, despite being fewer, may have superior functionality due to reduced exhaustion and improved antigen presentation by mature DCs. This is further supported by the unchanged number in the TME of cytotoxic CD8^+^ T cells, which are crucial for tumor killing. Moreover, the increased polarization of macrophages toward the M1 phenotype in the TME following RIPK3 activation (Figure 4F) suggests a decrease in antiinflammatory factors, which are often associated with tumor progression and immunosuppression (46). This alteration in the macrophage profile further contributed to an environment less favorable for tumor growth and more conducive to effective immune surveillance. These findings suggest that the RIPK3-driven system not only eliminated tumor cells as a safety switch but also reshaped the immune landscape of the TIME, fostering conditions that favor long-term immunity. This reshaping is essential not just for immediate tumor eradication but also for establishing a vigilant immune system capable of responding to future tumor challenges, thereby significantly reducing the risk of recurrence. This aligns with the growing recognition in the field of oncology that the key to effective cancer treatment lies not only in destroying tumor cells but also in reprogramming the immune system to prevent future occurrences.

TDLNs are the primary sites for the development of adaptive antitumor immunity, where activated DCs present tumor-related antigens to CD4^+^ and CD8^+^ T cells, leading to cytotoxic T cell activation and tumor clearance (24). While primary brain tumors in adults rarely metastasize distantly through lymphatic routes, recent research has revealed the existence of meningeal lymphatic vessels connecting the brain to the deep cervical lymph nodes (DCLNs) (47). Although tumor cells themselves may not readily metastasize through these channels, immune cells, antigens, and inflammatory mediators can be transported from the brain to the DCLNs, making them a critical site for studying tumor immunology and potential therapeutic targets. In our study, we focused on DCLNs as the primary TDLNs, observing an increase in DCs and NK cells after RIPK3 activation. These findings suggest that RIPK3 activation could potentially enhance the immunogenic capacity of TDLNs, thereby supporting antitumor immunity.

While our study focused on the efficacy of the RIPK3-driven necroptotic safety switch using whole living cancer cells, which pose more safety concerns than other therapeutic cell types, it is crucial to acknowledge the potential of this safety switch to be implemented in a vast array of cells used in immunotherapies. Cells such as T cells, NK cells, and mesenchymal stem cells, among others, play essential roles in immunotherapeutic strategies. Although the efficacy and safety of the RIPK3 system across these diverse cell types were not explored in our study, future research should encompass these cell types to provide a comprehensive understanding of the system’s applicability and effectiveness across the spectrum of cell-based immunotherapies.

Moreover, although our study successfully applied immune profiling to discern changes in immune cell population dynamics, it is important to acknowledge the limitations of this approach. Immune profiling provides a snapshot of the immune landscape, highlighting shifts in cell populations and their potential roles in the immune response. However, this method does not delve into the intricate molecular and cellular mechanisms driving these changes. For instance, while we observed an increase in the number of mature DCs in the TME after inducing RIPK3-driven cell death (Figure 4E), the signaling pathways, transcriptional changes, or intercellular interactions that led to this increase remain elusive. Understanding these underlying mechanisms is crucial, as they can offer insights into the broader implications of our findings, the potential therapeutic targets, and the nuances of the immune response. Future studies should aim to bridge this gap, integrating immune profiling with mechanistic investigations or other in-depth analyses to provide a better understanding of the effects observed in cell-based cancer immunotherapy.

In conclusion, our study’s introduction of the dual-function, RIPK3-driven necroptotic safety switch for cell-based immunotherapies reveals a promising avenue to address the safety concerns associated with these therapies as well as to synergistically enhance the therapeutic efficacy. By harnessing the immunogenic properties of necroptosis, this system not only ensures safety but also potentially amplifies the therapeutic benefits of cell-based immunotherapies.

## Methods

### Sex as a biological variable.

Previous studies on both mouse sexes have shown consistent results using the same cancer cell–based therapy. Therefore, our study was focused solely on female mice for easier handling and management purposes.

### Study design.

The objective of this study was to develop an efficient therapeutic strategy that could simultaneously induce a direct killing of tumor cells and elicit antitumor immune responses to counter the tumor aggressiveness as well as the immunosuppressive microenvironment. We hypothesized that genetic engineering of cancer cells would allow us to use and repurpose their self-homing and neoantigen-rich features for cancer treatment. All experiments performed in this study had at least 3 replicates to demonstrate biological reproducibility and to ensure adequate statistical power for comparisons. All animals were randomly allocated to the control or treatment group with equivalent tumor size. The study was not conducted in a blinded manner, and no statistical methods were used to predetermine the sample size. Details for in vivo experiments, number of cells used, duration, and statistical tests are described below as well as in the supplemental materials and figure legends.

### Intracranial deep implantation.

C57BL6 mice were anesthetized and immobilized on a stereotactic frame. Tumor cells (2 × 10^5^) in 3 μL PBS were implanted 2.2 mm deep, 2.5 mm lateral from bregma, and 2.5 mm ventral from dura in the left hemisphere for the first implantation and in the right hemisphere for the rechallenge. BLI was used to follow in vivo growth of Fluc-engineered implanted tumor cells over time using a PerkinElmer IVIS Lumina system. For Fluc imaging, mice were imaged 7 minutes after intraperitoneal injection of d-luciferin (122799, PerkinElmer). Animals were monitored closely and euthanized at the onset of any humane endpoints, including persistent recumbence, pain or distress that could be alleviated by analgesics, difficulty with ambulation, severe CNS signs, abnormal breathing and cyanosis, excessive weight loss, tumor production–specific endpoints, or model-specific endpoints. Animals were randomly allocated to cages and experimental groups.

### Mouse brain tissue and TDLN harvesting.

C57BL6 mice were perfused with 10 mL PBS by cardiac puncture. Tumor tissues from the brain were then harvested and processed for flow cytometric analysis. Intracranial injection of Evans Blue dye into brain TDLNs was performed, and subsequent surgical examination identified the DCLNs as the most proximal TDLNs. The DCLNs were also harvested for flow cytometric analysis.

### Cell lines.

The mouse GBM cell line CT2A was provided by I. Verma (Salk Institute for Biological Studies, La Jolla, California, USA). The mouse GBM cell line GL261 was provided by Sean Lawler (Brown University, Providence, Rhode Island, USA). All cells were cultured at 37°C in a humidified atmosphere with 5% CO_2_ and 1% penicillin/streptomycin (15140122, Invitrogen, Thermo Fisher Scientific) and grown in high-glucose DMEM (11965118, Invitrogen, Thermo Fisher Scientific) supplemented with 10% v/v FBS (A4766801, Invitrogen, Thermo Fisher Scientific). Cell lines were regularly tested for mycoplasma using a mycoplasma PCR kit (30-1012K, American Type Culture Collection [ATCC]).

### Determination of B/B, rapamycin, and GCV EC_50_.

The B/B homodimerizer (also called AP20187) was purchased from MilliporeSigma (SML2838). A stock solution was obtained by dissolving 5 mg lyophilized B/B in 100% ethanol at a concentration of 62.5 mg/mL and stored at –20°C. A working solution was then prepared for serial dilutions of B/B for its EC_50_ determination, at 100 nM, 50 nM, 1 nM, 0.5 nM, 0.25 nM, and 0.1 nM. Rapamycin was purchased from MedChem Express (HY-10219). Lyophilized rapamycin was dissolved in 6.25% DMSO in PBS at a concentration of 150 mM and stored at 4°C. A working solution was prepared for serial dilutions of rapamycin for its EC_50_ determination, at 100 nM, 50 nM, 25 nM, 20 nM, 15 nM, 10 nM, 5 nM, and 1 nM. GCV was purchased from MilliporeSigma (G2536). Lyophilized GCV was dissolved in 0.1 M HCl at a concentration of 10 mg/mL (10,000 μg/mL) and stored at –20°C. This solution has been used to perform serial dilutions of GCV for its EC_50_ determination, at 50, 25, 10, 5, 2.5, 1, 0.5, and 0.1 μg/mL. One day before treatment, 10,000 cells per well were plated in 96-well black plates and treated with different doses of B/B for 3 hours, rapamycin for 24 hours, or GCV for 72 hours. Cell death was observed under the microscope before performing the viability assay. For Fluc-expressing cells, the cells were then rinsed with 50 μL PBS per well before viability was measured using 50 μL d-luciferin (1:50) (122799, PerkinElmer) in PBS. Bioluminescence (BLU) was measured using a GloMax cell plate reader. Experiments were performed in triplicate.

### ATP assay.

A total of 80,000 cells were plated in 24-well plates and treated with EC_50_ doses of B/B, rapamycin, or GCV at different times to collect samples when approximately 25%, 50%, and 75% of the cells were dead. The supernatant was collected in microcentrifuge tubes and centrifuged at 9,248*g* for 15 minutes at 4°C. The supernatant was then transferred into new centrifuge tubes on ice. A standard curve ATP solution was made from a stock solution at 10 mM (20-306, MilliporeSigma). ATP from ATP standard curve solutions and the ATP within supernatant were then measured using a CellTiterGlo Luminescent Cell Viability Assay kit (G9241, Promega), diluted at 1:1 in FBS-free medium in a 96-well black plate, and read with a GloMax cell plate reader.

### Lentiviral transductions and engineering of stable cell lines.

Lentiviral packaging was performed by transfection of 293T cells using third-generation lentiviral systems with calcium chloride. Cells were cotransfected with 12 μg pRRL-EFS-MCS-FV-RIPK3-T2A-GFP lentiviral transfer plasmid and helper DNA (1.2 μg pCMV-VSVG, 2.4 μg pRSV-REV and 3.6 μg pMDLg/pRRE (gag and pol). After 16 hours, the medium was changed to half the original volume of fresh growth medium. Twenty-four hours after the medium change, the supernatant was collected without disturbing the cells, followed by centrifugation and filtering using a 0.45 μm PES syringe filter to collect and isolate viruses. Cells were transduced with unconcentrated lentiviral vectors in medium containing protamine sulfate (2 μg/mL) in 6-well plates (2 × 10^5^ cells per well). After expansion in 100 mm tissue culture dishes, CT2A-FmC RIPK3 cells were selected by FACS using a BD FACSAria Fusion cell sorter. For BLI, cancer cells were transduced with LV-Pico2-Fluc-mCherry and selected by FACS using a BD FACSAria Fusion cell sorter or by puromycin selection (1 μg/mL) in culture. GFP or mCherry expression was confirmed by fluorescence microscopy.

### Flow cytometry.

Tumor tissues from the brain or DCLNs were collected after perfusion with PBS and subsequently dissociated into a single-cell suspension through a 100 μm cell strainer (352360, Corning). Cell were counted and then resuspended in PBS before staining with viability dye using the Zombie UV Fixable Viability Kit (423107, BioLegend). Next, cells were washed with FACS buffer (2% BSA and 5 mM EDTA in PBS) and incubated with mouse FcR blocking reagent (130-092-575, Miltenyi Biotec) for 30 minutes. The samples were then stained with fluorophore-conjugated antibodies (Supplemental Table 3). Antibody dilutions were determined by titration with murine splenocytes. Following staining, samples were washed and fixed with either 2% PFA for extracellular staining or buffers from the True-Nuclear Transcription Factor Buffer Set (424401, BioLegend). Analysis was done using an LSR Fortessa Cytometer (BD) with BD FACSDiva and FlowJo (version 10) software with the gating strategy shown in Supplemental Figure 1. Compensation was done with antibody-stained UltraComp eBeads (01-2222-41, Thermo Fisher Scientific) and calculated using FACSDiva software.

### Western blot analysis.

After treatment, cells were washed twice with cold PBS and then lysed with cold RIPA buffer (20 mM Tris-HCl pH 8.0, 137 mM NaCl, 10% glycerol, 1% NP-40, 0.1% SDS, 0.5% Na-deoxycholate, 2 EDTA pH 8.0) supplemented with protease and phosphatase inhibitors (Roche protease inhibitor cocktail and Phosphatase Inhibitor Cocktail I and Phosphatase Inhibitor Cocktail II from MilliporeSigma). Cells were then centrifuged at 4°C, 16,000*g* for 10 minutes. Supernatant protein concentrations were determined using a Bio-Rad protein assay kit. 6× SDS-sample buffer was added to the samples, which were then boiled for 3 minutes and resolved on SDS-PAGE gels. Protein (10–40 μg) was resolved on an SDS-PAGE gel, transferred onto a nitrocellulose membrane, and probed with primary antibodies. Antibodies against p-MLKL (37333, Cell Signaling Technology), p-RIPK3 (91702S, Cell Signaling Technology), caspase3 (9662S, Cell Signaling Technology), cleaved caspase 3 (9661S, Cell Signaling Technology), HGMB1 (3935S, Cell Signaling Technology), and vinculin (V4505, MilliporeSigma) were used for Western blot analysis. HRP-conjugated secondary antibodies against rabbit (97023, Abcam) were used for all primary antibodies, except vinculin, for which HRP-conjugated secondary antibodies against mice were used (97051, Abcam).

### TCGA analysis.

The mRNA expression profile and patient information regarding tumor samples were extracted from the R2 Genomics Analysis Visualization Platform (https://hgserver1.amc.nl.cgi-bin/r2/main.cgi). Extracted data were entered into Excel and Cluster 3.0 to generate graphs and heatmaps. The signature score was calculated by taking the average value of the *z* score for each gene in each sample.

### CCGA analysis.

The mRNA expression profile and patient information regarding the tumor samples were extracted from the Chinese Glioma Genome Atlas (CGGA) (http://ccga.org.cn). Extracted data were entered into Excel and Cluster 3.0 to generate graphs and heatmaps. The signature score was calculated by taking the average value of the *z* score for each gene in each sample.

### Statistics.

Data are expressed as the mean ± SD for in vitro studies and as the mean ± SEM for in vivo studies and were analyzed by an unpaired, 2-tailed Student’s *t* test (Figure 1, C and F) or 2-way ANOVA with the 2-stage step-up method of the Benjamini, Krieger, and Yekutieli test for multiple-comparison correction (Figure 4, D–H). Survival times of mouse groups were analyzed and compared using a log-rank (Mantel-Cox) test with Bonferroni correction (Figure 1, B, E, and L; Figure 3, C, E, and F; Figure 4C; Figure 5, C, E, G, and I). GraphPad Prism 9 (GraphPad Software) was used for all statistical analysis and also to generate Kaplan-Meier survival plots. A *P* value of less than 0.05 was considered statistically significant. Experiments were performed with at least 3 replicates to demonstrate reproducibility and to ensure adequate statistical power for comparisons. The sample sizes for each group for each experiment are shown in the figure legends. All mice were randomly assigned to the various experimental groups, and all samples were analyzed equally. Exclusion criteria were not preestablished, and no sample or data points were omitted from analysis.

### Study approval.

All in vivo procedures were approved by the IACUC of Brigham and Women’s Hospital.

### Data availability.

All data are available in the main text or the supplemental materials. All supporting data values are available in the Supporting Data Values file.

## Author contributions

KS and KSC conceptualized the study. KSC, SMB, NK, IV, YCC, LYL, FR, PB, SJK, NCM, and KS designed the study methodology. KSC, SMB, and KS conducted experiments. KSC, SMB, and KS performed visualization. FKS acquired funding. KSC and KS were responsible for project administration. KSC and KS supervised the study. KSC, SMB, and KS wrote the original draft of the manuscript. KSC, FR, and KS reviewed and edited the manuscript.

## Supplementary Material

Supplemental data

Unedited blot and gel images

Supplemental table 1

Supplemental table 2

Supplemental table 3

Supporting data values

## Version 1 11/19/2024

In-Press Preview

## Version 2 01/16/2025

Electronic publication
