# Supplementary material for: An inducible RIPK3-driven necroptotic system enhances cancer cell–based immunotherapy and ensures safety
Source: J Clin Invest. 2024 Nov 19;135(2):e181143. doi: 10.1172/JCI181143 (PMC11735097; doi:10.1172/JCI181143)
Supplement: Supplemental data [file jci-135-181143-s239.pdf]

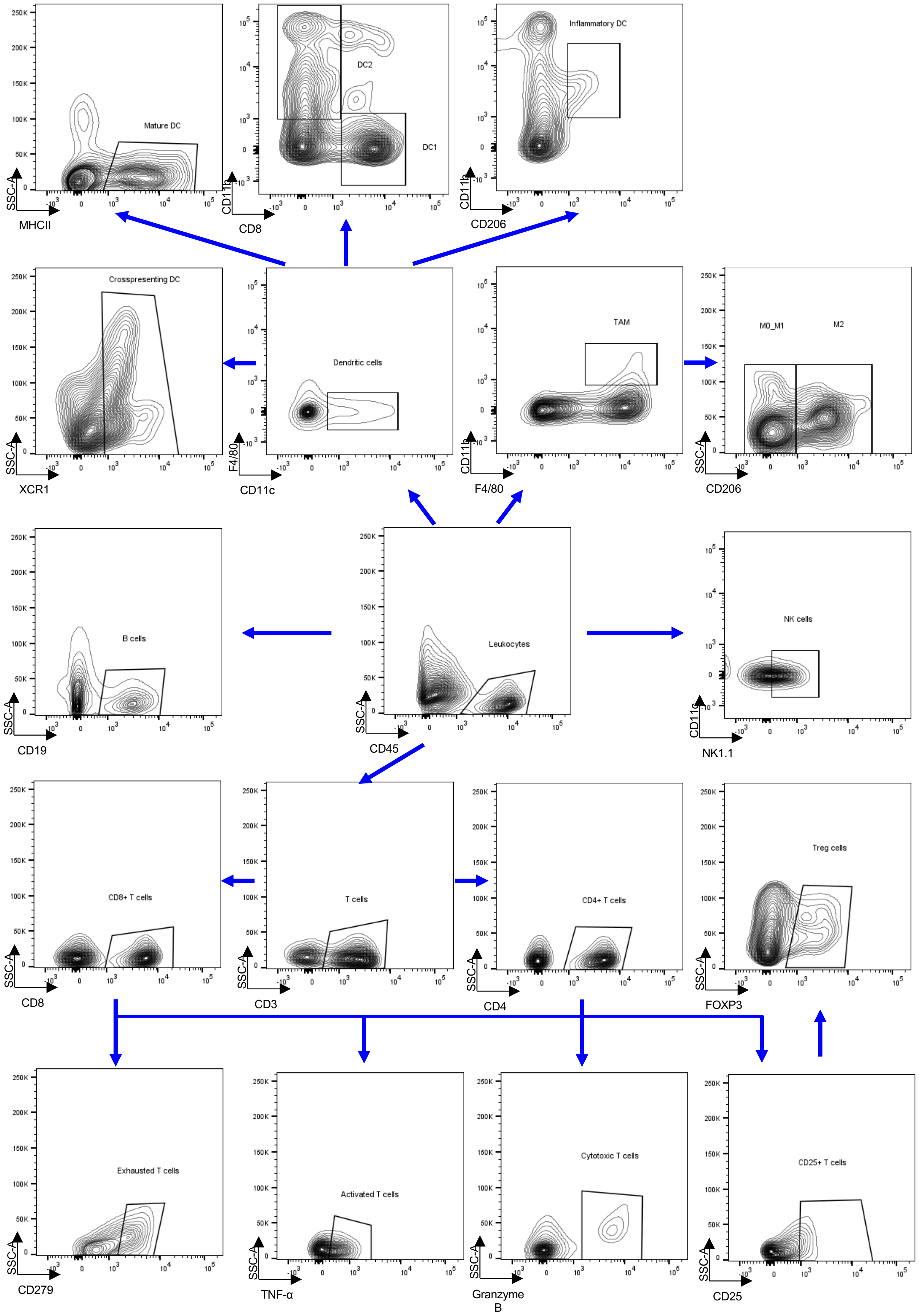

Mature DC
SSC-A
MHCII
DC2
DC1
CD11b
CD8
Inflammatory DC
CD11b
CD206
Crosspresenting DC
SSC-A
XCR1
Dendritic cells
F4/80
CD11c
TAM
CD11b
F4/80
M0_M1
M2
SSC-A
CD206
B cells
SSC-A
CD19
Leukocytes
SSC-A
CD45
NK cells
CD11c
NK1.1
CD8+ T cells
SSC-A
CD8
T cells
SSC-A
CD3
CD4+ T cells
SSC-A
CD4
Treg cells
SSC-A
FOXP3
Exhausted T cells
SSC-A
CD279
Activated T cells
SSC-A
TNF-α
Cytotoxic T cells
SSC-A
Granzyme B
CD25+ T cells
SSC-A
CD25

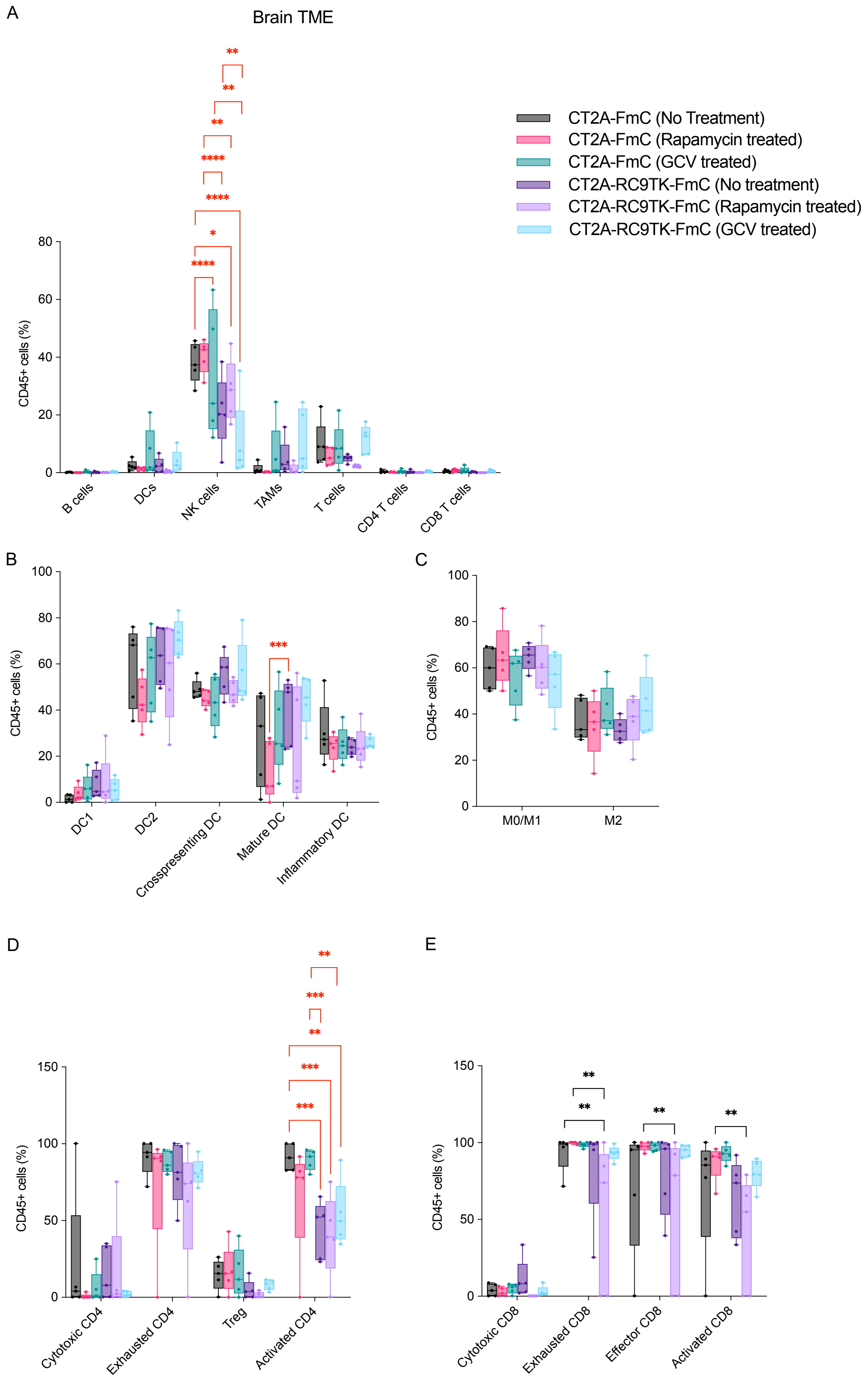

A
Brain TME
CT2A-FmC (No Treatment)
CT2A-FmC (Rapamycin treated)
CT2A-FmC (GCV treated)
CT2A-RC9TK-FmC (No treatment)
CT2A-RC9TK-FmC (Rapamycin treated)
CT2A-RC9TK-FmC (GCV treated)
CD45+ cells (%)
B cells
DCs
NK cells
TAMs
T cells
CD4 T cells
CD8 T cells
B
C
DC1
DC2
Crosspresenting DC
Mature DC
Inflammatory DC
M0/M1
M2
D
E
Cytotoxic CD4
Exhausted CD4
Treg
Activated CD4
Cytotoxic CD8
Exhausted CD8
Effector CD8
Activated CD8

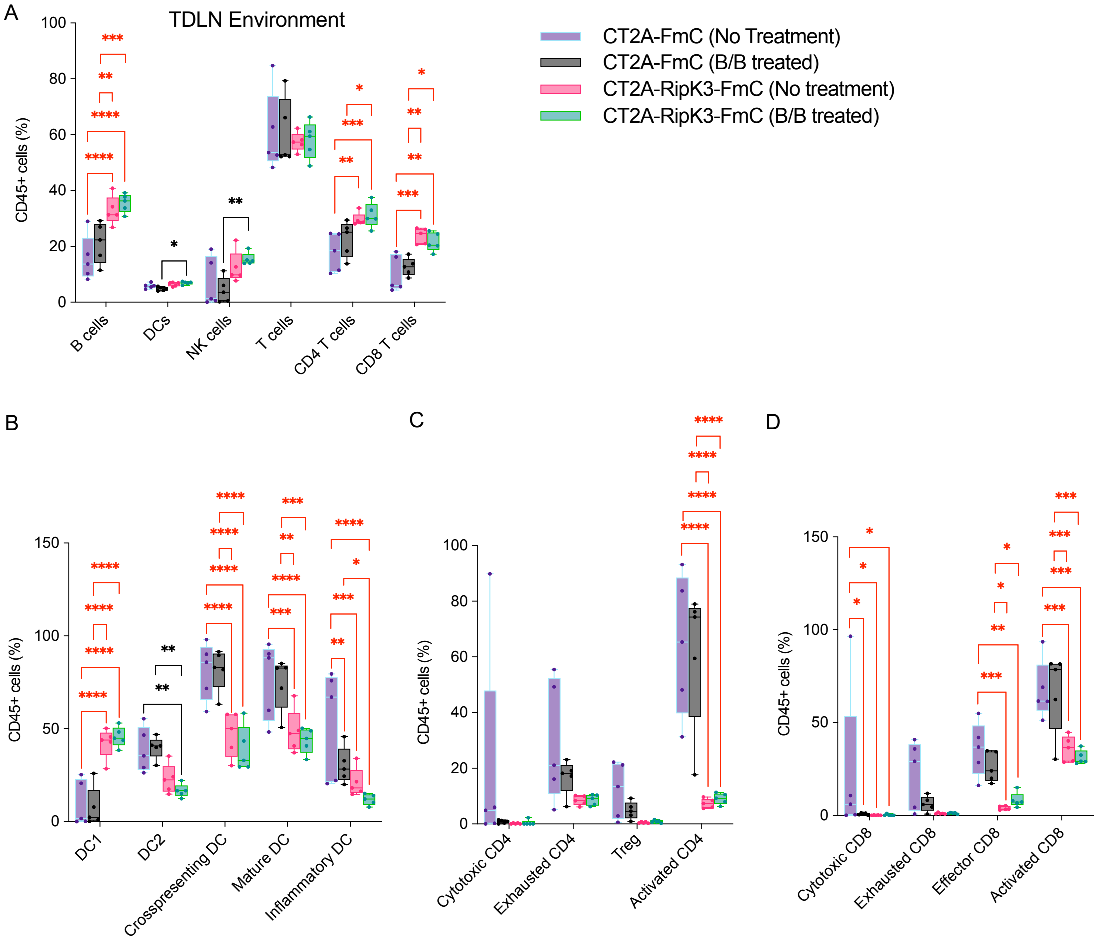
A
TDLN Environment
CT2A-FmC (No Treatment)
CT2A-FmC (B/B treated)
CT2A-RipK3-FmC (No treatment)
CT2A-RipK3-FmC (B/B treated)
CD45+ cells (%)
B cells
DCs
NK cells
T cells
CD4 T cells
CD8 T cells
B
DC1
DC2
Crosspresenting DC
Mature DC
Inflammatory DC
C
Cytotoxic CD4
Exhausted CD4
Treg
Activated CD4
D
Cytotoxic CD8
Exhausted CD8
Effector CD8
Activated CD8

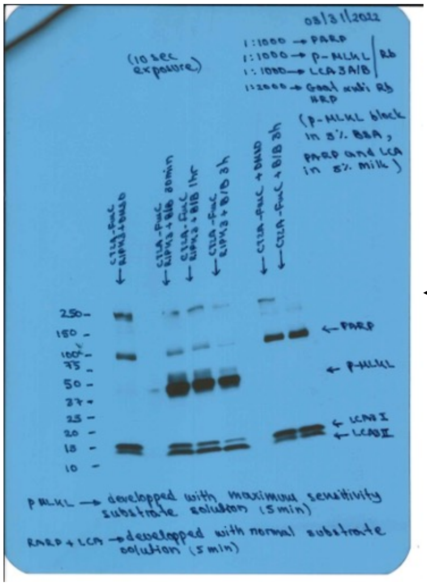
03/31/2022
(10 sec exposure)
1:1000 → PARP
1:1000 → p-MLKL
1:1000 → LC3A/B
1:2000 → Goat anti Rb HRP
(p-MLKL block in 5% BSA, PARP and LC3 in 5% milk)
250
150
100
75
50
37
25
20
15
10
← PARP
← p-MLKL
← LC3 I
← LC3 II
pMLKL → developped with maximum sensitivity substrate solution (5 min)
PARP + LC3 → developped with normal substrate solution (5 min)

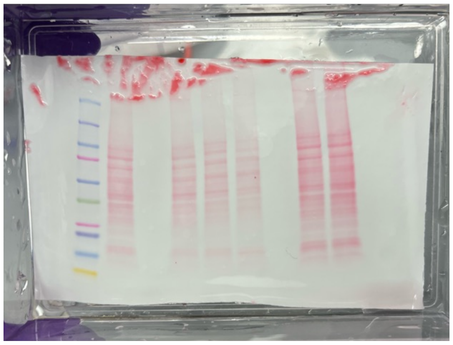

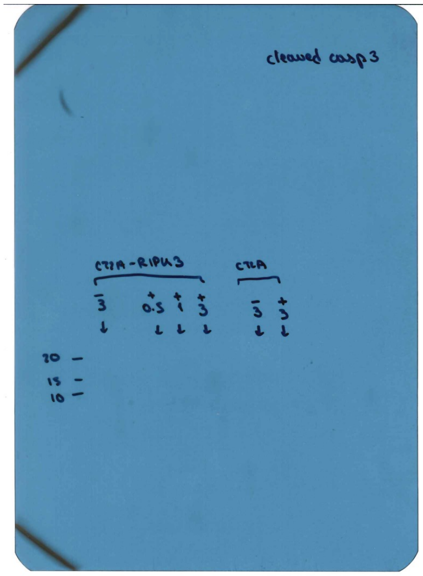
cleaved casp3
C71A-RIPK3
C71A
3
0.5
1
20
15
10

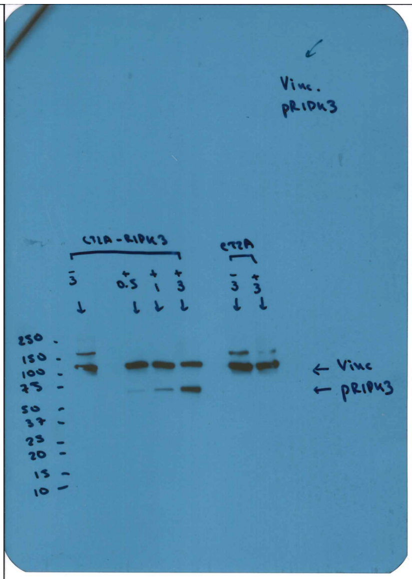
Vinc.
pRIPK3
C71A-RIPK3
C71A
250
150
100
75
50
37
25
20
15
10
← Vinc
← pRIPK3

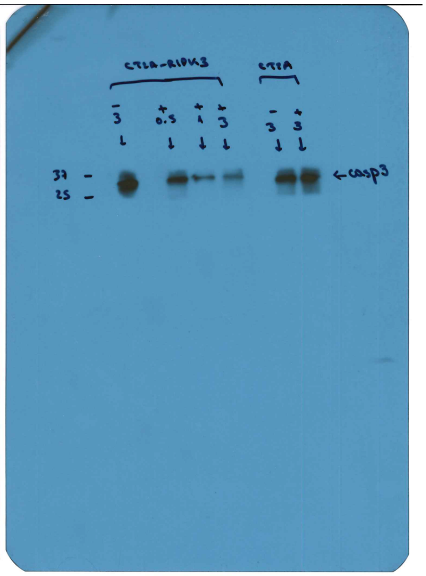
C71A-RIPK3
C71A
37
25
← casp3

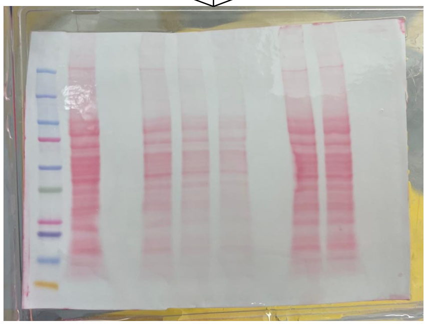

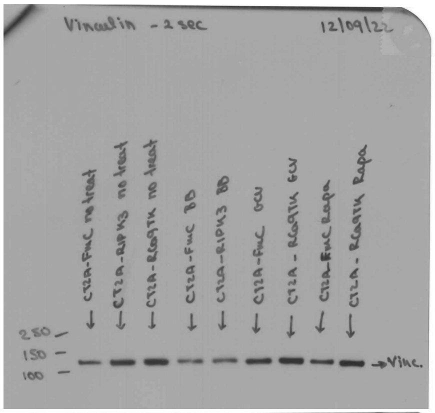
Vinculin - 2 sec
12/09/22
C12A-FUC no treat
C12A-RIPK3 no treat
C12A-RC4ATK no treat
C12A-FUC BB
C12A-RIPK3 BB
C12A-FUC GCU
C12A-RC4ATK GCU
C12A-FUC Rapa
C12A-RC4ATK Rapa
250
150
100
Vinc.

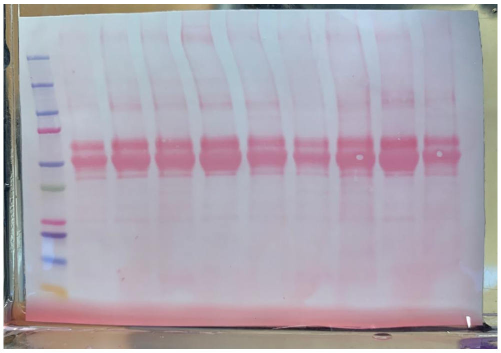

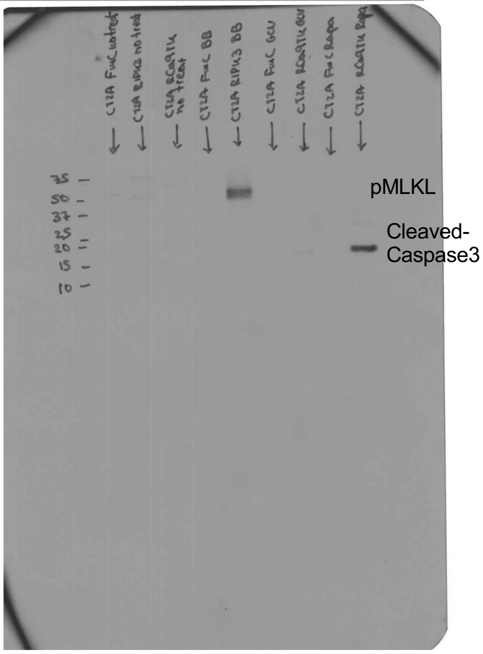
75
50
37
25
20
15
10
pMLKL
Cleaved-
Caspase3

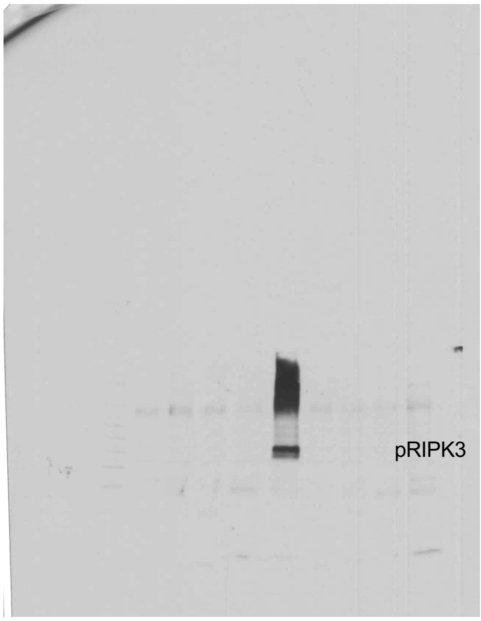
pRIPK3

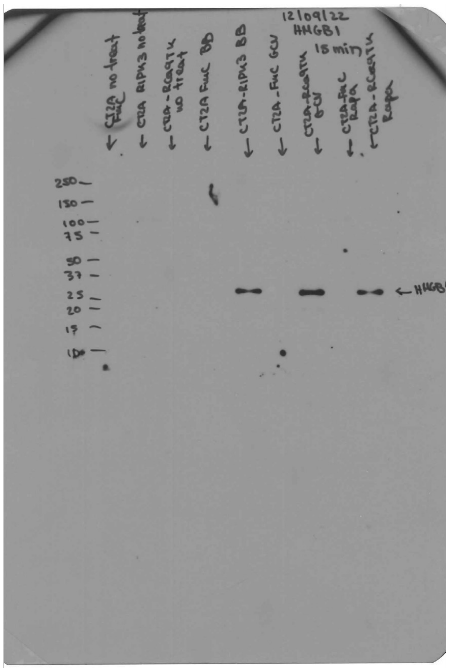
12/09/22
HMGB1
15 min
250
150
100
75
50
37
25
20
15
10

Supplementary Figures and Legends

Figure. S1 Flow plots demonstrate the gating strategy for immune profiling on tissues harvested from the mouse brain and TDLN.

Figure. S2 Cell death induced by RapaCas9 and HSV-TK safety switches has minimal effects on tumor immune microenvironment in vivo. (A) Boxplot showing immune profiling of tumor tissues harvested from the mouse brain 5 days post prodrug treatment for the groups of CT2A with rapamycin treatment (n=5), GCV treatment (n=5), and without any treatment (n=5), and CT2A-RC9TK with rapamycin treatment (n=5), GCV treatment (n=5), and without any treatment (n=5) (B) Boxplot showing the profile of DC subpopulations from the experiment in (A). (C) Boxplot showing the profile of TAM subpopulations from the experiment in (A). (D) Boxplot showing the profile of CD4 T subpopulations from the experiment in (A). (E) Boxplot showing the profile of CD8 T subpopulations from the experiment in (A). The whiskers represent minimum to maximum. Data were analyzed by 2-Way ANOVA with the two-stage step-up method of Benjamini, Krieger and Yekutieli test for multiple comparison correction. * = $P<0.05$, ** = $P<0.01$, *** = $P<0.001$. The significance line in black represents changes due to the activation of the suicide system, whereas in red represents changes due to differences in cell type or drug.

Figure. S3 Cell death induced by RIPK3 safety switch reinvigorates TDLN environment in vivo. (A) Boxplot showing immune profiling of TDLN harvested from the mice 5 days post prodrug treatment for the groups of CT2A with (n=5) or without (n=5) B/B treatment and the groups of the mixture of CT2A and CT2A-RIPK3 with (n=5) or without (n=5) B/B treatment. (B) Boxplot showing the profile of DC subpopulations from the experiment in (A). (C) Boxplot showing the profile of CD4 T subpopulations from the experiment in (A). (D) Boxplot showing the profile of CD8 T subpopulations from the experiment in (A). The whiskers represent minimum to maximum. Data were analyzed by 2-Way ANOVA with the two-stage step-up method of Benjamini, Krieger and Yekutieli test for multiple comparison correction. * = $P<0.05$, ** = $P<0.01$, *** = $P<0.001$. The significance line in black represents changes due to the activation of RIPK3 safety switch, whereas in red represents changes due to differences in cell type or drug.

Figure. S4 Unedited blots for Fig. 1I.

Figure. S5 Unedited blots for Fig. 2F.
