# Supplementary figures and images for: An inducible RIPK3-driven necroptotic system enhances cancer cell–based immunotherapy and ensures safety
Source: J Clin Invest. 2024 Nov 19;135(2):e181143. doi: 10.1172/JCI181143 (PMC11735097; doi:10.1172/JCI181143)

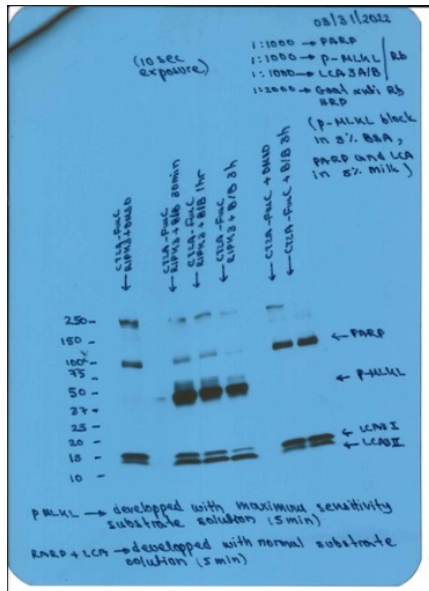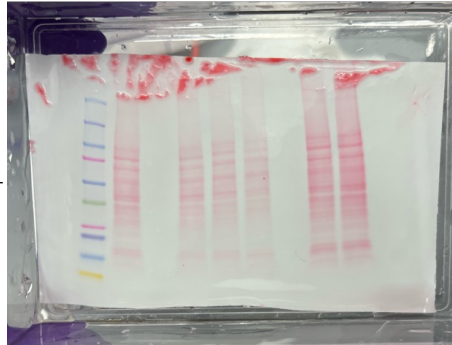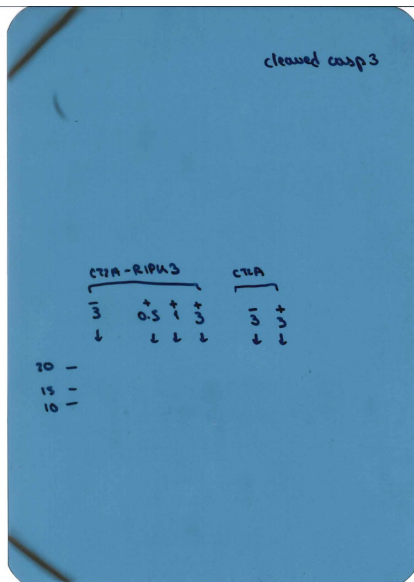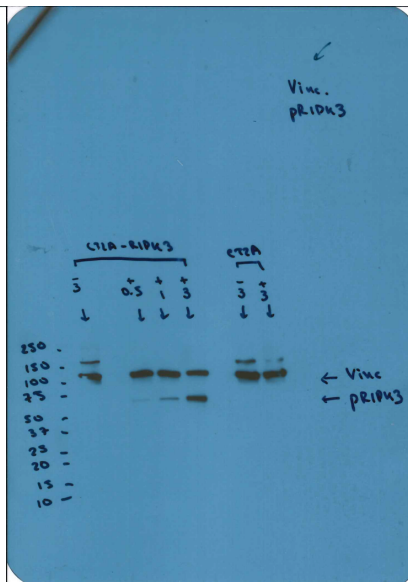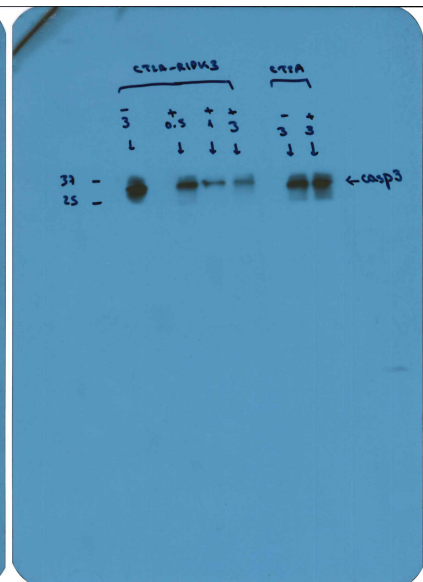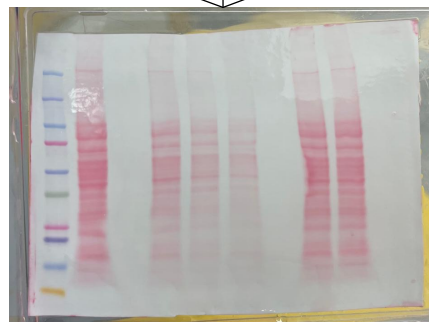

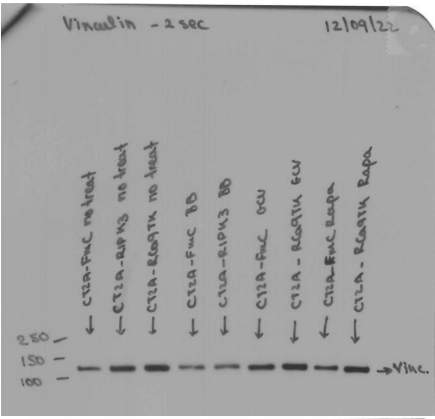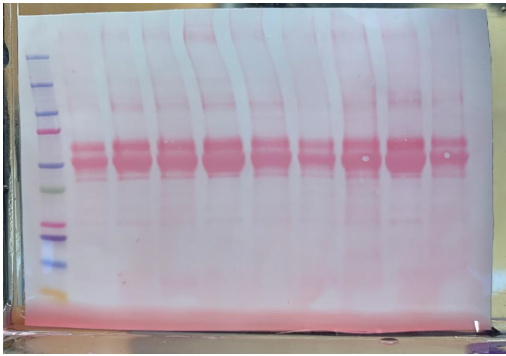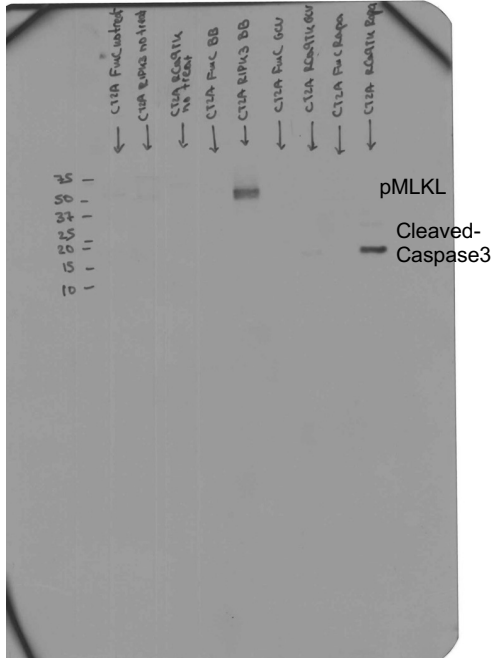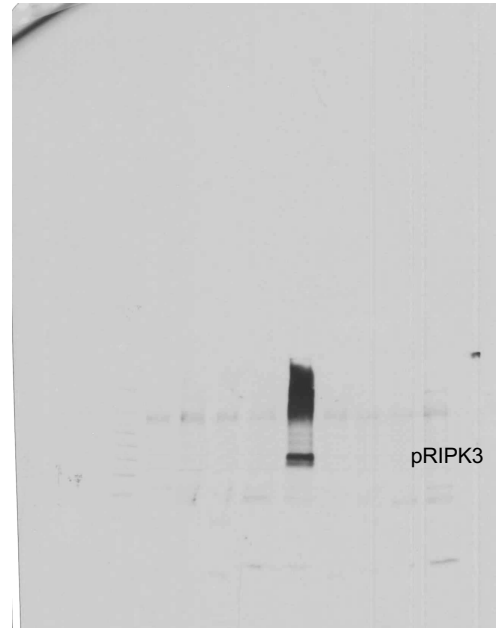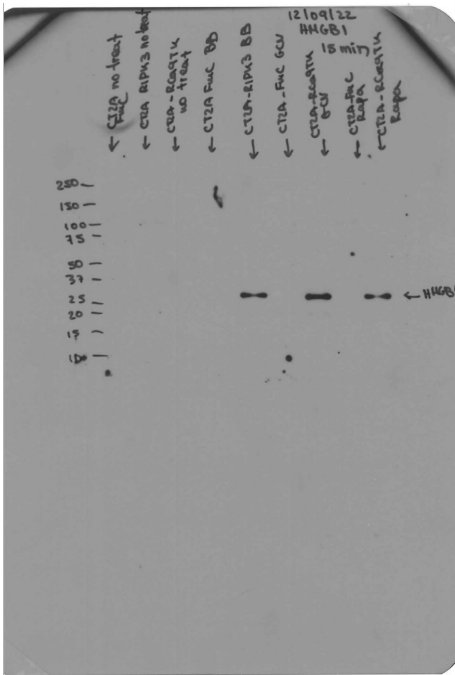

Supplement: Unedited blot and gel images [file jci-135-181143-s240.pdf]
